# Genetics of Myelodysplastic Syndromes

**DOI:** 10.3390/cancers13143380

**Published:** 2021-07-06

**Authors:** Caner Saygin, Lucy A. Godley

**Affiliations:** Section of Hematology/Oncology, Department of Medicine, The University of Chicago, 5841 S. Maryland Ave., MC 2115, Chicago, IL 60637, USA; caner.saygin@uchospitals.edu

**Keywords:** myelodysplastic syndrome, genetics, germline predisposition, clonal hematopoiesis

## Abstract

**Simple Summary:**

Myelodysplastic syndrome (MDS) is a disease of the bone marrow, characterized by an inability of the bone marrow to produce normal numbers of peripheral blood cells. There are several different types of MDS, and these are driven by distinct biological pathways. The past several years have seen great advances in developing new treatment strategies based on this molecular understanding of the disease drivers. Thus, accurate diagnosis and effective treatment now rely on an accurate assessment of each patient’s particular disease. Most exciting now is the recognition of precursor conditions that may allow strategies to delay or even prevent MDS development altogether.

**Abstract:**

Myelodysplastic syndrome (MDS) describes a heterogeneous group of bone marrow diseases, now understood to reflect numerous germline and somatic drivers, characterized by recurrent cytogenetic abnormalities and gene mutations. Precursor conditions including clonal hematopoiesis of indeterminate potential and clonal cytopenia of undetermined significance confer risk for MDS as well as other hematopoietic malignancies and cardiovascular complications. The future is likely to bring an understanding of those individuals who are at the highest risk of progression to MDS and preventive strategies to prevent malignant transformation.

## 1. Introduction

Both germline and somatic variants contribute to the development of myelodysplastic syndrome (MDS), a heterogeneous group of bone marrow malignancies resulting in ineffective hematopoiesis and bone marrow failure, with risk of progression to acute myeloid leukemia (AML) [1,2]. To help highlight some specific features that are important to consider at the time of MDS diagnosis and throughout management, we will begin by describing a representative case that reflects how patients present to their health care provider: A 62-year-old woman came to the Hematology clinic for evaluation of pancytopenia. Ten years ago, she was diagnosed with stage III estrogen-receptor-positive breast cancer and treated with surgery, radiation, and adjuvant chemotherapy using adriamycin, cyclophosphamide, and paclitaxel. She has been in remission from her breast cancer since completion of chemo/radiotherapy. She has no other medical problems. At presentation, her complete blood count showed a total white blood cell count of 2200/µL, with an absolute neutrophil count of 1300/µL, hemoglobin of 11.9 g/dL, and a platelet count of 87,000/µL. She had 1% circulating blasts. A bone marrow biopsy demonstrated 15% cellularity with trilineage dysplasia and 7% blasts. Cytogenetic analysis revealed trisomy 8, and a next generation sequencing (NGS) molecular profiling panel showed a DDX41 c.1496dup (p.Ala500Cysfs*9) mutation at a variant allelic frequency (VAF) of 48%, and an ASXL1 c.1934dup (p.G646Wfs*12) mutation with a VAF of 23%. She was diagnosed with a therapy-related myelodysplastic syndrome (MDS) with excess blasts, which was stratified as intermediate-risk based on a Revised International Prognostic Scoring System (R-IPSS) score of 4.5. She began therapy with 5-azacitidine, given for 7-days subcutaneously every 28 days.

## 2. Diagnosis of MDS

MDSs are a heterogenous group of myeloid neoplasms characterized by cytopenias, varying degrees of dysplasia, and a risk of progression to acute myeloid leukemia (AML) [1,2]. The median age at diagnosis is 76 years [3]. The clinical presentation of MDS ranges from indolent conditions with minimal symptoms and mild cytopenias to subtypes that are more comparable to AML. This clinical heterogeneity has long been recognized, and MDSs were classified into different subtypes based on clinical, morphologic, and genetic characteristics. However, this classification was not adequate for prognostication and treatment selection, leading to the development of the International Prognostic Scoring System (IPSS) [4]. This risk stratification classification system is based on bone marrow blast percentage, cytogenetic abnormalities, and number of cytopenias. IPSS has been adopted for use in MDS clinical trials, and subsequently was revised in 2012 as R-IPSS by accounting for the severity of each cytopenia and expanding the genetic risk profile [5]. Since that time, our knowledge of the biology and genetics of MDS has improved with widespread adoption of NGS, which has enabled the identification of recurrent gene mutations throughout disease evolution [6,7,8,9,10]. As the functional consequences of these mutations are characterized, new prognostic systems and therapeutic approaches have been proposed, promising a brighter future for MDS treatment and prevention [11].

Bone marrow failure is the hallmark of MDS, which is caused by the selective growth advantage of mutated hematopoietic stem and progenitor cell (HSPC) clones within a supportive microenvironment. In MDS patients, the bone marrow is normo- or hypercellular in 85% of cases, whereas 15% of patients have hypoplastic MDS (hMDS) [12]. The latter is more common in MDS patients with certain germline mutations, as well as in patients who have a T-cell mediated autoimmunity against their HSPCs [13]. MDS can be distinguished from aplastic anemia and other myeloid neoplasms based on the presence of >10% dysplasia in one or more cell lineages, and by MDS-associated karyotypic abnormalities, such as del(5q), monosomy 7, and trisomy 8 [14].

Below, we provide an overview of the genetics of MDS, focusing on the impact of somatic mutations and germline predisposition. We highlight the recently recognized precursor conditions, clonal hematopoiesis of indeterminate potential (CHIP) and clonal cytopenia of undetermined significance (CCUS), and the interplay between genetic and environmental factors in disease evolution.

## 3. Somatic Mutation Landscape in MDS

### 3.1. Cytogenetic Abnormalities

**Figure 1:**
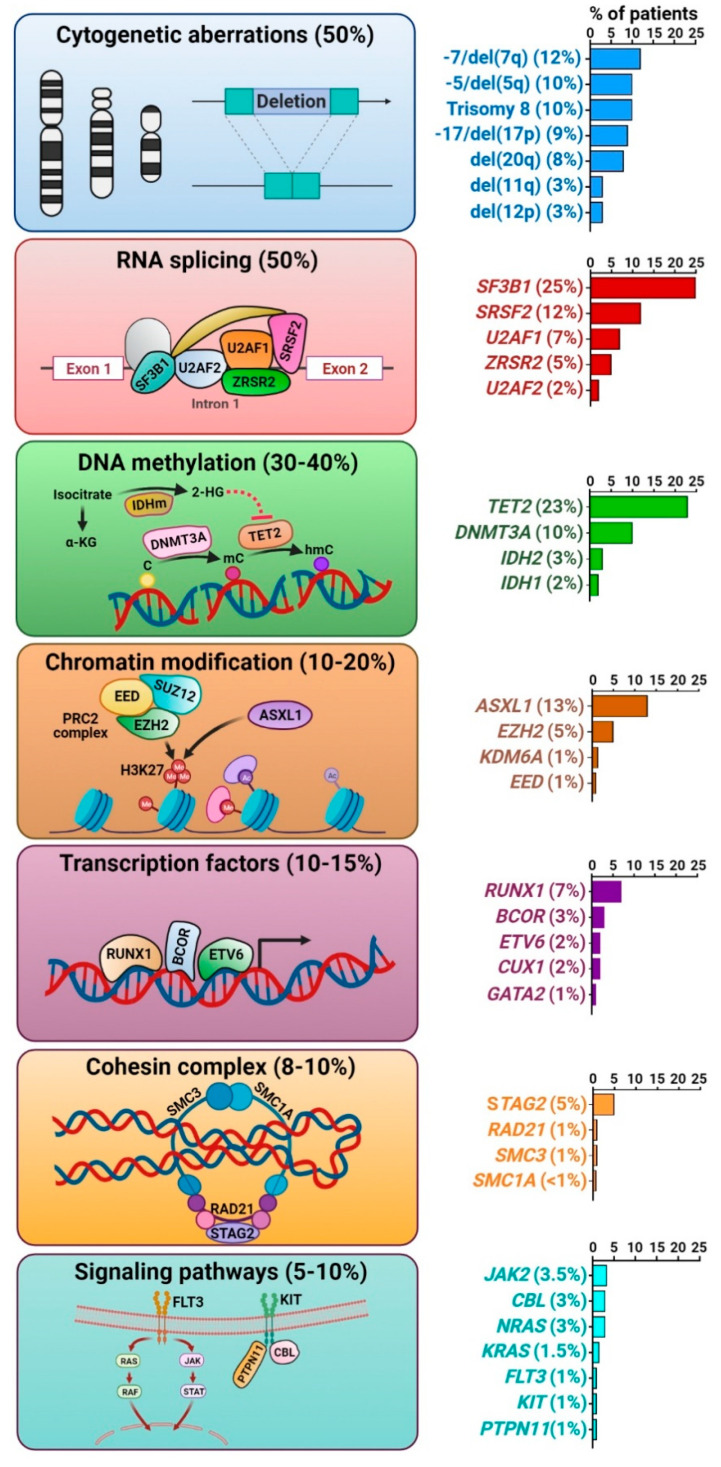
Somatic mutation landscape in myelodysplastic syndromes. Schemas highlight individual groups of mutations, and the associated bar graphs on the right provide the mutation percentages.

Early studies in MDS genetics focused on acquired cytogenetic abnormalities detected by conventional karyotyping, which are present in ~50% of MDS patients [15]. Most of these abnormalities are unbalanced chromosomal alterations, resulting in loss or gain of genetic material. The most frequent karyotypic abnormalities include −5/del(5q), −7/del(7q), trisomy 8, del(11q), del(12p), −17/del(17p), del(20q), and +21q (Figure 1). These are distinct from the cytogenetic changes commonly found in AML, which are typically balanced translocations, such as t(8;21)(q22;q22), inv(16)(p13q22), and 11q23 rearrangements, all of which encode fusion oncoproteins that drive leukemogenesis [16]. In MDS, many of these unbalanced karyotypic abnormalities co-occur within complex karyotypes (≥3 abnormalities). Recurrent reciprocal translocations are rare (<3% of cases), but may be associated with characteristic morphologic abnormalities, such as abnormal megakaryocytes and thrombocytosis, as seen with inv(3)(q21q26) or t(3;3)(q21;q26). Although not routinely tested clinically, single-nucleotide polymorphism (SNP) array-based platforms can detect copy number alterations (CNAs) as well as copy-neutral loss-of-heterozygosity (CN-LOH) or uniparental disomy [17]. These are commonly seen in chromosomes where recurrently mutated myeloid genes reside, such as 1p (*MPL* and *NRAS*), 4q (*TET2*), 7q (*CUX1* and *EZH2*), 11q (*CBL*), 13q (*FLT3*), and 17p (*TP53*) [18].

### 3.2. Somatic Gene Mutations

Whole exome sequencing technologies have enabled detection of recurrent somatic mutations in more than 50 genes in 80–90% of MDS cases [6,7]. In a patient with MDS, the median number of somatic mutations within the coding sequence is 9 [1]. This is substantially lower than the number of mutations seen in solid tumors. Furthermore, the average number of mutations varies depending on the disease subtype: 6 per exome in low-risk MDS, 9 in MDS with excess blasts, 8 in MDS/myeloproliferative neoplasm (MPN) unclassified, and 12 in chronic myelomonocytic leukemia [19]. The target driver gene mutations can be categorized into distinct functional pathways, including those that encode proteins that are important in the spliceosome complex (e.g., SF3B1, SRSF2, U2AF1, and ZRSR2), DNA methylation (e.g., DNMT3A, TET2, and IDH1/2), chromatin/histone modification (e.g., ASXL1 and EZH2), cohesin complex (e.g., RAD21, STAG2, SMC1, and SMC3), myeloid transcription factors (e.g., RUNX1, GATA2, CUX1, BCOR, and ETV6), tumor suppressors (e.g., TP53 and PHF6), and signaling pathways (e.g., KIT, RAS, FLT3, and CBL) [6,7,8,9]. Most of these genes are mutated in <5% of cases, and no single mutation accounts for the majority of cases, which contributes to MDS genetic and clinical heterogeneity. Although there is significant overlap in the somatic gene mutations seen in MDS compared to de novo AML, the frequencies at which those mutations are seen differ. Mutations involving receptor tyrosine kinases (such as FLT3), RAS pathway, CEBPA, and IDH are more common in AML, whereas mutations in the spliceosome complex and epigenetic regulators are more frequent in MDS.

When multiple genetic changes are present, there are clinically significant patterns of co-occurrence or mutual exclusivity among different mutations. For example, *TP53* mutations are often associated with complex karyotypes, and this combination portends a poor prognosis and inferior response to chemotherapy [20]. Mutations involving spliceosome complex components are heterozygous and mutually exclusive of each other, likely due to synthetic lethality of multiple mutations in this pathway [21]. A similar pattern has also been shown for cohesin complex mutations [22]. This likely points to the vitality of these proteins for cell viability, such that loss of all copies is not compatible with survival, which could be exploited therapeutically to target cells with specific combinations of somatic mutations. This mechanism was promising in preclinical work on spliceosome pathway inhibitors, and those are now being investigated in early-phase studies [23].

#### 3.2.1. Splicing Factor Mutations

RNA splicing factor mutations represent the most prevalent somatic gene mutations in MDS, of which the most commonly affected are those in the U2RNP complex, including SF3B1, SRSF2, U2AF1, and ZRSR2, and less commonly, SF1, U2AF2, and SF3A1. These mutations lead to widespread RNA splicing alterations, causing mis-splicing of key hematopoietic regulators via alternative 5′- or 3′-splice site usage, reduced or enhanced intron retention, and inclusion or exclusion of cassette exons [24]. The gene most commonly mutated in MDS is *SF3B1*, found in 25% of patients and specifically associated with ring sideroblasts, which confers a favorable prognosis [25]. *SF3B1* mutations alter the function of the encoded splicing factor, promoting alternative 3′-splice site usage in *ABCB7* and other genes involved in mitochondrial iron transportation and causing abnormal iron deposition around mitochondria to form ring sideroblasts [21,26]. Mutations in *SRSF2* and *U2AF1* are also missense and gain-of-function, often causing alternative exon usage. On the contrary, *ZRSR2* mutations on the X chromosome are nonsense or frameshift, leading to protein inactivation [21]. This causes retention of minor (U12-type) introns, which makes up <1% of the introns in humans [27]. Targets of mutant splicing factor proteins implicated in MDS pathogenesis include ERFE, BRD9, MAP3K7, PDS5A, and NF1 for mutated *SF3B1*, and EZH2, CDK10, and CASP8 for mutated *SRSF2* [26,28]. Therefore, splicing factor mutations exert differential effects on global RNA splicing and contribute to leukemogenesis via unique mechanisms.

#### 3.2.2. Epigenetic Regulator Mutations

Methylation of CpG islands within gene promoters is a major epigenetic control mechanism for regulation of transcription. This is frequently dysregulated in MDS and AML with changes in methylation patterns effecting both CpG islands in the gene promoter regions, as well as intragenic and intergenic regions, including enhancers [16]. The most frequently mutated genes include *DNMT3A*, *TET2*, *IDH1*, and *IDH2*. *DNMT3A* is involved in de novo DNA methylation of cytosine to methylated cytosine at CpG residues. In the presence of α-ketoglutarate (α-KG), TET2 catalyzes the conversion of methylated cytosine to 5′-hydroxymethylcytosine, which can act as an activating epigenetic mark or as an intermediate in a demethylation pathway [18,29]. IDH1 and IDH2 are isoforms of the isocitrate dehydrogenase enzyme localized in cytoplasm and mitochondria, respectively. Each catalyzes the conversion of isocitrate to α-KG, required by many cellular enzymes, including the TETs. Mutant IDH1 and IDH2 enzymes gain neomorphic enzyme activity causing conversion of α-KG to 2-hydroxyglutarate, which in turn inhibits cellular reactions that require α-KG as substrate, including TET2 and histone deacetylases such as KDM6A [30]. Mutations in *IDH* and *TET2* are mutually exclusive, suggesting a shared pro-leukemogenic effect [7,9].

#### 3.2.3. Chromatin and Histone Modifier Mutations

Genes involved in polycomb-related proteins are frequently mutated in myeloid neoplasms. There are two major polycomb group complexes in humans, polycomb repressive complex 1 (PRC1) and 2 (PRC2), which ubiquitinate and methylate histones, respectively [31]. *EZH2* encodes a component of PRC2 and is mutated in ~5% of MDS patients [9]. These mutations are characterized by loss-of-function, leading to reduced global H3K27me3 levels and deregulated gene expression [32]. Similarly, loss-of-function mutations in genes encoding other PRC2 components (e.g., *EED* and *SUZ12*) deregulate normal hematopoiesis by derepression of the key genes in HSPCs [33]. *ASXL1* is the most frequently mutated gene in this category and its encoded protein physically interacts with the PRC2 complex. When mutated or truncated, ASXL1 inhibits PRC2-mediated trimethylation of H3K27 [31]. Mutations involving *ASXL1* and *EZH2* are associated with a poor prognosis in MDS and AML [34,35].

#### 3.2.4. Transcription Factor Mutations

Another class of major mutational targets in MDS is myeloid transcription factors, which regulate gene expression by binding to specific DNA sequences. Common targets include *RUNX1*, *ETV6*, and *GATA2* [7]. Mutations in these genes are seen as somatic events in 10–15% patients, but they can also be found as germline variants, discussed in more detail below. RUNX1 is a master transcription factor controlling HSPC differentiation and plays critical roles in embryogenesis and adult hematopoiesis [36]. Together with other transcription factors, such as GATA proteins, RUNX1 regulates key genes in hematopoiesis, such as *KIT* [37]. *RUNX1* is often co-mutated with other classes of mutations, such as *ASXL1* and *STAG2* in MDS [38].

#### 3.2.5. Cohesin Complex Mutations

The cohesin ring complex consists of SMC1A, SMC3, RAD21, and STAG proteins, which are recruited to chromatin in concert with cohesin-associated proteins, such as ESCO, NIPBL, and CTCF, and regulate gene expression through the formation of large-scale chromatin structures that maintain the three-dimensional genome architecture, control sister chromatid cohesion during cell division, and facilitate DNA repair [39]. Mutations in these genes are found in ~10% of MDS cases, leading to loss-of-function and altered chromatin structures that allows for accessibility to a number of transcription factors, including RUNX1 and GATA2 [22,40].

#### 3.2.6. Transcription Factor Mutations

In comparison to AML, mutations involving signaling pathways are less common in MDS, each representing <5% of cases [9]. These include *JAK2*, *CBL*, *RAS*, *FLT3*, *KIT*, and *PTPN11* mutations. Notably, *JAK2* mutations and, less commonly, *MPL* and *CALR* mutations are often found in MDS/MPN with ring sideroblasts and thrombocytosis, which is a distinct MDS/MPN overlap entity [2].

## 4. Case Continued

Our patient started therapy with 5-azacitidine for her R-IPPS intermediate-risk MDS with trisomy 8 and frameshift mutations in *DDX41* and *ASXL1*. After four cycles of treatment, her bone marrow biopsy showed 20% cellularity with 1% blasts and no evidence of dysplasia. Cytogenetic analysis revealed a normal karyotype and NGS showed disappearance of the *ASXL1* mutation, but persistence of the *DDX41* c.1496dup (p.Ala500Cysfs*9) mutation at a VAF of 49%. The persistence of the *DDX41* mutation raised concern for germline predisposition [41], especially considering this specific variant is seen as a germline allele most commonly in Asian populations [42]. Importantly, her family history was significant for AML in her mother and a maternal aunt. Although her hematologist recognized the potential germline nature of this allele, she had a difficult time identifying a clinical laboratory that could test *DDX41* as a germline predisposition gene, since the commercial laboratory contracted by her practice did not cover this gene in its inherited leukemia panel [43]. Once an appropriate laboratory was identified, a skin biopsy was performed, and DNA extracted from cultured fibroblasts demonstrated the same *DDX41* frameshift mutation as detected in her MDS cells, confirming the germline origin of this deleterious variant. During the evaluation of potential related donors for an allogeneic hematopoietic stem cell transplant (HSCT), her brother was identified as HLA-identical and lacking the deleterious *DDX41* germline variant. Therefore, she underwent allogeneic HSCT with reduced intensity conditioning, using her brother’s peripheral blood stem cells. She remains in remission at 14 months post-transplant.

## 5. Germline Predisposition to MDS

The identification of the *DDX41* p.A500fs variant should immediately raise concerns about germline predisposition, since it is a well-recognized germline allele within Asian populations [44]. However, many clinicians have a misunderstanding that germline genetics are not relevant to patients who present with MDS in the typical elderly age range [45]. In fact, the average age of diagnosis of a myeloid malignancy in those with a germline *DDX41* mutation is 65–70 years old, the same as the general population [46,47]. Clinicians also may not appreciate that since germline alleles are present in all cells of an individual’s body, even malignant cells contain germline variants. Panels designed to provide prognostic information about hematopoietic malignancies that cover genes mutated in both somatic and germline settings (e.g., *CEBPA*, *GATA2*, *RUNX1*, and *TP53*) will reveal germline variants, even if that is not the primary intention of the test [48,49]. It is critical for accurate diagnosis and patient management that clinicians recognize the potential germline nature of deleterious variants and pursue appropriate follow-up studies to test the germline status of such variants [48,49,50,51]. *DDX41* variants found on somatic AML/MDS NGS panels with a variant allele frequency (VAF) of > 40% have been confirmed in the germline in 94% of patients, particularly early truncating variants as well as germline variants common in particular populations (e.g., those encoding p.M1? and p.D140fs in Northern Europeans; and, as noted above, p.A500fs in Asians) [44]. Important to also recognize is that the somatic hotspot variant p.R525H is found in more than half of the patients with germline *DDX41* variants [44]. In myeloid malignancies, patients with a ‘double-hit’ in a gene such as *CEBPA*, *RUNX1*, or *DDX41* often have one variant in the germline with a VAF between 40 and 60%, and a second variant of somatic origin with variable VAF [52].

To date, the strongest associations between germline predisposition mutations and development of hematopoietic malignancies are seen for MDS. Germline *SAMD9*/*SAMD9L* mutations are seen in young children [53,54], and about 15% of adolescents and young adults, and up to 40% of patients with monosomy 7, have germline *GATA2* mutations [55,56]. In adults diagnosed with MDS from 18 to 40 years old, 19% have germline mutations, commonly in DNA repair and telomere biology genes [57]. In older adults with MDS, germline *DDX41* mutations predominate [42,46,47,58,59]. Thus, the age at which an individual presents with MDS can almost predict the gene in which a deleterious germline variant will be identified. We recommend germline testing when (1) a deleterious variant is found in a gene known to confer a germline risk for a myeloid malignancy at a VAF >30%; (2) the variant is truncating and/or affects well-described germline hotspots; and/or (3) there is a ‘double-hit’ with one of the variants being within germline range. Special consideration should be given to deleterious variants in genes such as *CEBPA*, *DDX41*, *ETV6*, *GATA2*, *RUNX1*, and *TP53*, or those associated with telomere biology disorders [44,48,49,50,51,60,61,62,63,64,65].

Although somatic NGS panels can identify germline variants, they should not be used in standard screening and testing, since somatic panels do not include all of the genes associated with germline predisposition syndromes or non-coding genomic loci known to contain germline hotspots. Moreover, somatic reversion occurs frequently in hematopoietic tissues, resulting in failure to identify germline mutations in blood and bone marrow. Germline confirmation of any variant detected via somatic testing should be performed on true germline DNA, which can be obtained from cultured skin fibroblasts, bone marrow-derived mesenchymal stromal cells, or hair roots [57,66,67]. Segregation of a gene variant among family members is another way of demonstrating an allele’s germline status. Thus, the tissues that are most often collected from an MDS patient, including blood, bone marrow, and saliva, all contain hematopoietic cells, and are therefore often contaminated with malignant cells [68].

Most individuals and families are identified with germline predisposition to hematopoietic malignancies when careful attention is paid to the personal and family history of the MDS patient along with the molecular profiling data. Key features of the initial clinical presentation that signal a likelihood of having germline predisposition include a personal history of two or more cancers; personal history of a hematopoietic malignancy along with a family history of another hematopoietic malignancy/prolonged cytopenia/or other hematologic abnormality, such as macrocytosis, or onset of a non-hematopoietic tumor at an age <50 years old within two generations of the proband; and/or molecular testing of malignant cells showing a deleterious variant in a gene known to confer cancer risk, especially when that variant persists despite a change in disease status, such as from diagnosis through remission, as our clinical case demonstrates.

## 6. Evolution of MDS Clones from Precursor Conditions

**Figure 2:**
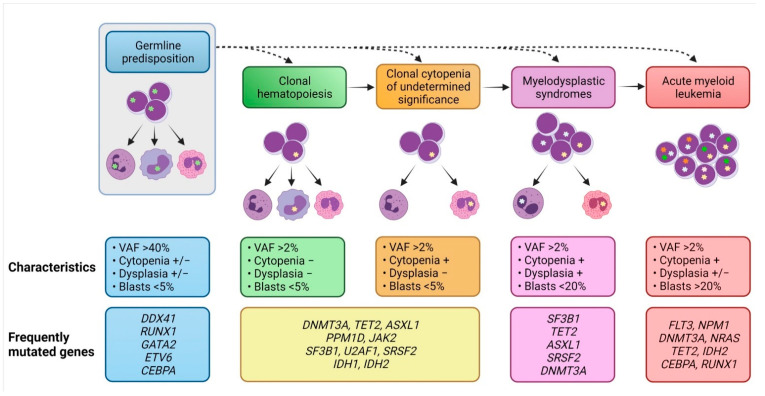
Evolution of myeloid malignancies from their antecedent precursor lesions. Germline mutations predispose individuals to acquire somatic mutations in their hematopoietic stem cells, leading to clonal hematopoiesis (CH) as well as clonal cytopenia of undetermined significance (CCUS). Both CH and CCUS are precursor conditions with increased risk for myelodysplastic syndrome (MDS) and acute myeloid leukemia (AML). Clinical characteristics, diagnostic criteria, and the most frequently affected genes are highlighted for each condition.

It has long been recognized that AML may arise from antecedent MDS, or patients with AML who do not have a pre-existing MDS diagnosis may have evidence of significant dysplasia in their bone marrow (>50% in at least two lineages), classified as AML with myelodysplasia-related changes [2]. Recently, hematopoietic precursor lesions with increased risk for MDS and AML have been described in persons with cytopenias, as well as in those with normal blood counts (Figure 2).

The presence of somatic myeloid mutations at a VAF ≥ 2% with no hematologic compromise defines a pre-malignant condition called clonal hematopoiesis of indeterminate potential (CHIP) [69,70]. CHIP is rare at a young age, but its prevalence increases to ~10% among individuals aged >70 years and continues to increase as the population ages. The most frequently mutated genes in CHIP include *DNMT3A*, *TET2*, *ASXL1*, *PPM1D*, *U2AF1*, *SF3B1*, *SRSF2*, *TP53*, *JAK2*, *CBL*, and *IDH2* [69]. These mutations are also common in patients with MDS and AML, with the exception of *PPM1D*. Clonal hematopoiesis has also been detected by CNAs, overlapping with those karyotypic abnormalities seen in MDS and AML, such as trisomy 9, del(11q), del(13q), and del(20q) [71,72,73]. In these studies, both myeloid mutations and CNAs were associated with a 10- to 30-fold increased risk for development of myeloid malignancies [71,72]. When cytopenias accompany mutations in blood cells, this condition is classified as CCUS [70]. The risk for myeloid neoplasms is even higher for these individuals [74]. Collectively, these observations suggest that MDS and AML may be initiated by CHIP-related mutations or chromosomal lesions. However, the overall risk for developing a myeloid neoplasm is low (1% per year on average), and most of these older individuals do not develop MDS or AML in their lifetime. Therefore, it is important to evaluate each case individually by factoring the type of mutation(s), size(s) of the clone(s), prior history of genotoxic therapies, comorbidities, and average life expectancy. In a cohort of individuals with de novo CHIP, all participants with *TP53* and *IDH* mutations progressed to develop AML [75]. The median time to AML was 4.9 years with *TP53*-mutated CHIP, but more than 5 years with *IDH*-mutated CHIP. Other high-risk CHIP mutations include *U2AF1*, *SF3B1*, and *SRSF2* [76]. The presence of more than one mutation, as well as the presence of a mutation at VAF > 10% further increase the risk for MDS/AML [76]. Ongoing research aims to identify the factors associated with the development of specific CHIP clones and progression of pre-existing clones (e.g., genotoxic therapies or smoking), in order to develop the appropriate preventive strategies for individuals at high risk of AML and MDS [77,78].

## 7. Conclusions

MDS is characterized by genetic and biologic heterogeneity, which creates unique challenges in developing new therapeutics. Disease occurs when somatic mutations accumulate in HSPCs, which is a result of environmental exposures (e.g., genotoxic therapies) in a conducive bone marrow microenvironment. When this process occurs in a genetically predisposed individual, MDS occurs in much younger individuals than the average. Identification of pre-MDS lesions (CHIP and CCUS) has spawned the development of specific attention to people with these conditions to monitor them for the development of blood cancers and adverse cardiovascular outcomes. The far-reaching goal is to identify individuals with the highest risk of developing a myeloid malignancy and to intervene to prevent malignant progression. Our efforts are in their infancy, and there is much more to learn about CHIP as we aspire towards preventative strategies in hematologic malignancies.
